# Podocytes as novel sources of neutrophil serine proteases: expression and regulation by inflammatory molecular patterns

**DOI:** 10.1007/s00018-025-06014-y

**Published:** 2025-12-15

**Authors:** Aleksandra Wróblewska-Schmude, Irena Audzeyenka, Wiktoria Mallek, Magdalena Wysocka, Adam Lesner, Magdalena Narajczyk, Felicja Gajdowska, Danuta Gutowska-Owsiak, Tomasz Kulesza, Dorota Rogacka, Agnieszka Piwkowska

**Affiliations:** 1 Laboratory of Molecular and Cellular Nephrology, Mossakowski Medical Research Institute, Polish Academy of Sciences, Gdansk, Poland; 2 Doctoral School of Translational Medicine, Medical Center of Postgraduate Education, Warsaw, Poland; 3 Department of Biomedical Chemistry, Faculty of Chemistry, University of Gdansk, Gdansk, Poland; 4 Department of Environmental Technology, Faculty of Chemistry, University of Gdansk, Gdansk, Poland; 5 Department of Electron Microscopy, Faculty of Biology, University of Gdansk, Gdansk, Poland; 6 Experimental and Translational Immunology Group, Intercollegiate Faculty of Biotechnology of University of Gdansk and Medical University of Gdansk, University of Gdansk, Gdansk, Poland; 7 Laboratory of Experimental and Translational Allergology and Pneumonology, Medical University of Gdańsk, 80--210, Gdańsk, Poland; 8 Laboratory of Molecular Enzymology and Oncology, Intercollegiate Faculty of Biotechnology, University of Gdansk and Medical University of Gdansk, Gdansk, Poland

**Keywords:** Podocyte, Elastase, Proteinase 3, Cathepsin G, Extracellular vesicles, Myeloperoxidase, Oxidative stress

## Abstract

Podocytes are essential components of the glomerular filtration barrier and are increasingly recognized as immunologically active cells. Here, we demonstrate that human and rat podocytes express enzymatically active neutrophil serine proteases (NSPs), including neutrophil elastase, proteinase 3, and cathepsin G, as well as their endogenous inhibitors, serpins. We show that the expression and activity of these proteases are regulated by pathogen- and damage-associated molecular patterns. Notably, podocytes release NSPs in extracellular vesicles and secrete mitochondrial DNA in response to inflammatory stimuli without compromising cell viability. We also identified, for the first time, the expression and redistribution of myeloperoxidase in podocytes upon stimulation. These findings reveal a previously unrecognized role of podocytes, suggesting that they may actively participate in glomerular inflammation and immune responses. The present study provides new insights into podocyte biology and opens avenues for exploring their role in kidney disease pathogenesis.

## Introduction

The recruitment of immune cells to the glomerulus is a time-consuming process. Neutrophils infiltrate the glomerulus within a few hours of the initiating stimulus, reaching their peak at approximately 24 h [1, 2]. Infiltration of the glomerulus with reactive oxygen species (ROS)-producing macrophages results in a further increase in oxidative stress and leads to the activation of podocyte apoptosis [3]. Considering that people with diabetes are particularly susceptible to infections and that podocytes are exposed to pathogens during the process of blood filtration, it is not surprising that these cells have developed several features of immune cells [4, 5]. These features allow podocytes to quickly protect themselves against threats and metabolic imbalance; for example, they express certain genes that are characteristic of immune cells, including B7-1 (CD80, a costimulatory molecule required for T-cell activation) [6] and FcRn (the neonatal Fc receptor involved in IgG transfer) [7]. Podocytes are equipped with several families of pattern recognition receptors, such as Toll-like receptors [8–10], NOD-like receptors [11, 12], and NACHT, LRR, and PYD domain-containing protein (NLRP) inflammasomes [13, 14], which translate the recognition of danger into the secretion of proinflammatory cytokines and chemokines. Moreover, activated podocytes release factors that directly stimulate neutrophil chemotaxis, promote secretory vesicle exocytosis, and prime neutrophils for increased ROS generation. The impact of the neutrophil granule content on podocyte cytoskeleton disruption and the loss of podocyte integrity was demonstrated, contributing to the development of proteinuria in nephrotoxic nephritis [15]. Neutrophil activation also leads to the release of multiple cytotoxic products, including ROS, neutrophil extracellular traps (NETs), and proteinases. Neutrophil elastase (NE), proteinase 3 (PR3), cathepsin G (CG), and NSP4 are neutrophil serine proteinases (NSPs) that are stored in high concentrations within azurophilic (primary) granules; these enzymes, in combination with ROS, contribute to the degradation of engulfed microorganisms inside the neutrophil’s phagolysosomes [16]. These proteinases are also externalized in their active form during neutrophil activation at inflammatory sites, contributing to the regulation of inflammatory and immune responses [17]. Upon their stimulation by inflammatory mediators, neutrophils exhibit increased release of extracellular vesicles (EVs) and altered EV composition [18, 19]. Moreover, these EVs can contain bioactive molecules, including neutrophil serine proteases [20–22]. Through EV-mediated transport, NSPs can remain protected from circulating inhibitors and retain their proteolytic potential in the extracellular environment [21]. This mechanism enables the transfer of active proteases and signalling molecules to other cells, potentially influencing podocyte function, glomerular permeability, and the propagation of inflammatory responses within the kidney.

Experimental studies have demonstrated that the production of oxygen radicals and the activity of lysosomal enzymes are important mediators of glomerular capillary wall injury. Proteinase 3, NE, and myeloperoxidase (MPO) have been detected in tubular casts and within tubular epithelial cells (TECs) in renal biopsies from patients with Wegener’s granulomatosis [23]. The authors suggested that PR3 released by polymorphonuclear neutrophils is taken up by renal parenchymal cells [23]. Subsequent studies have shown that TECs and glomerular epithelial cells express both the PR3 protein and mRNA in vitro in response to proinflammatory cytokines [24–26]. However, the presence of PR3 in nonhematopoietic cells remains controversial [27].

Recently, we demonstrated that podocytes express and secrete cathepsin C (CatC) [28]. We showed that a hyperglycemic environment increased CatC levels, enzymatic activity, and secretion into the extracellular space. Moreover, we confirmed that elevated CatC expression in podocytes was correlated with an increase in glomerular albumin permeability [28]. These findings suggest a novel mechanism of podocyte injury in diabetes and provide further insights into the role of CatC in the regulation of kidney function. Additionally, the most well-known function of CatC is the activation of immune cell-associated serine proteases, such as NSPs.

The present study sought to determine whether podocytes are able to express neutrophil serine proteases (NSPs) and their inhibitors, serpins, and to investigate how their expression may be regulated by signaling pathways dependent on molecular patterns such as pathogen-associated molecular patterns (PAMPs) and damage-associated molecular patterns (DAMPs). We sought to fill a critical gap in our understanding of the immunological capabilities of podocytes. The present findings may significantly advance our knowledge of podocyte biology, revealing previously unrecognized mechanisms by which these cells participate in immune responses and maintain glomerular barrier integrity. Ultimately, this research could redefine the role of podocytes as not only structural but also immunologically active components of the kidney.

## Materials and methods

### Immortalized human podocytes

The immortalized human podocyte cell line (CIHP‑1; RRID: CVCL_W186; from Prof. Moin A. Saleem, University of Bristol, UK [29]) was cultured in RPMI 1640 (Thermo Fisher Scientific, MA, USA) medium supplemented with 1% v/v antibiotics (penicillin [100 U/ml]/streptomycin [100 µg/ml] [P/S]; Sigma Aldrich, MO, USA) and 10% fetal bovine serum (FBS, Thermo Fisher Scientific, MA, USA). The cells were allowed to proliferate at 33 °C to 60% confluence and then differentiated at 37 °C for 10‒20 days in an atmosphere of 95% air/5% CO_2_. The expression of podocyte markers (podocin, synaptopodin, and nephrin) was determined via immunofluorescence staining. The following compounds were used in the experiment: LPS (1 µg/ml, 24 h; catalogue no. L5418, Sigma-Aldrich, MO, USA), ATP (100 µM, 30 min; catalogue no. 5.05419, Merck, MA, USA), H_2_O_2_ (100 µM, 15 min; catalogue no. 1.07298, Merck, MA, USA) and PMA (100 nM, 24 h; catalogue no. sc-3576B, Santa Cruz Biotechnology, TX, USA).

### Ethics statement and isolation of primary rat podocytes

The experiments were performed with Wistar rats obtained from the Mossakowski Medical Research Institute, Polish Academy of Sciences, Warsaw, Poland. The rats were maintained on a 12 h/12 h light/dark cycle with free access to a standard pellet diet and tap water. All experimental procedures involving animals were reviewed and approved in compliance with Directive 2010/63/EU of the European Parliament and Council on the Protection of Animals Used for Scientific Purposes. The ARRIVE guidelines were employed for reporting experiments that involved live animals, promoting ethical research practices. All experiments were conducted in compliance with the guidelines of the University of Gdańsk (4/D000/2024).

We used female Wistar rats weighing 100–120 g. After the animals were immobilized via a dorsal grip, they were administered a mixture of ketamine (65 mg/kg body weight) and xylazine (5/mg/kg body weight) intraperitoneally. The kidneys were excised and minced with a scalpel and then pressed through a series of sieves with decreasing pore diameters (160, 106, and 53 μm) to obtain a suspension of glomeruli in RPMI 1640 supplemented with 10% FBS and 100 U/ml penicillin with 100 µg/ml streptomycin. The final suspension of glomeruli was plated on 75 cm^2^ type I collagen-coated culture flasks (Becton Dickinson Labware, Beckton, UK) and maintained at 37 °C in an atmosphere of 95% air and 5% CO_2_ for 5‒7 days. Podocytes were isolated as described previously [30]. Experiments were conducted using podocytes that were cultivated for 12–20 days. Cell phenotypes were established using podocyte-specific antibodies against nephrin and synaptopodin.

### Lentiviral transduction

A human podocyte cell line with silenced ELANE, the gene encoding neutrophil elastase, was generated. Cells were transduced with GIPZ ELANE short-hairpin RNA (shRNA) viral particles (shELANE) or GIPZ non-silencing shRNA viral particles (Control; Dharmacon, Inc. CO, USA), which served as a negative control. To enhance transduction efficiency, polybrene (catalogue no. H9268; Sigma-Aldrich, MO, USA) was added to actively dividing podocytes. Puromycin (catalogue no. P9620, Sigma Aldrich, MO, USA) selection was subsequently applied to obtain stable shRNA-expressing podocyte populations.

### RNA isolation and real-time PCR

Cellular RNA from podocytes was isolated via the RNeasy Mini Kit (Qiagen, Hilden, Germany) according to the manufacturer’s recommendations. The quantity and purity of the isolated RNA were assessed via spectrophotometric measurements (NanoDrop 2000; Thermo Fisher Scientific, MA, USA). Reverse transcription was performed using 1500 or 3000 ng of RNA, dNTPs (10 mM), dT_15_ (0.5 µg), RNase inhibitor (28 U), and reverse transcriptase M-MLV (200 U). The expression of specific genes was analysed via real-time PCR, which was performed in a LightCycler 480 (Roche, Canton Zug, Switzerland) using specific intron-spanning primers and dual-labelled hydrolysis probes. The results were calculated via the ∆∆Ct method, with β-actin used as a control. The amplified products were separated on a 2.5% agarose gel and imaged with a GelDoc-It Imaging System (UVP, Cambridge, UK).

To assess the levels of mtDNA and nucDNA in cfDNA, SybrGreen-based real-time PCR was performed via gene-specific primers for the tRNA_Leu gene (mtDNA) and β2 microglobulin gene (nucDNA). Each reaction contained 31.5 ng of total cfDNA. Real-time PCR analyses were performed in a LightCycler 480 (Roche). The results were quantified via the ΔΔCt method. The primers and probes used are listed in Table 1.

**Table 1:** Primers and probes used in real-time PCR

Gene	Accession no.	Primer sequences (5’-3’)	Probe sequences (5’-3’)	Product (bp)
*hPRTN3*	NM_002777.3	Forward: CAGGAGCTCAATGTCACC Reverse: GAGTCTCCGAAGCAGATG	5’-FAM- TGGTCACCTTCTTCTGCCGG-BHQ-1	98
*hELANE*	NM_001972.3	Forward: GTGGCGAATGTAAACGTC Reverse: CTGGAGAATCACGATGTC	5’-FAM- CCTGGGAGCCCATAACCTCTC-BHQ-1	153
*hCTSG*	NM_001911.2	Forward: CCACAATATCCAGAGACG Reverse: CTGCTCAGCTGCAATAAC	5’-FAM-AAACACCCAGCAACACATCACT -BHQ-1	120
*hSERPINA3*	NM_001085.5	Forward: ACAAGATGGAGGAAGTGGAAGC Reverse: CACTAATGCAGAAAGGAGGGTGA	5’-FAM-GCCAGGAA-BHQ-1	317
*hSERPINB1*	NM_030666.4	Forward: GCATATGGCTACATCGAGGAC Reverse: GCGAGGTCGGAGTTGAGA	5’-FAM- TGCCGTGTGCTGGAACTGCCTTACC-BHQ-1	254
*hSERPINE1*	NM_000602.5	Forward: CAGCTCATCAGCCACTGGAA Reverse: CACCGTGCCACTCTCGTT	5’-FAM- CCCGCCTCCTGGTTCTGCCCAAGTTCT-BHQ-1	234
*hAKT*	NM_001101.5	Forward: ATTGGCAATGAGCGGTTC Reverse: GGATGCCACAGGACTCCA	5’-FAM-AGGCACTCTTCCAGCCTTC-BHQ-1	76
*rPRTN3*	NM_001024264.2	Forward: TGGAATCCGCCCATCCCTCAAA Reverse: GGGTCTCCTCGGGGTTGTAAT	5’-FAM-ACCTGCTG -BHQ-1	243
*rELANE*	NM_001106767.1	Forward: AGCACTGGCCTCAGAGATTG Reverse: ATGGTAGCTGAGCCATTGAGC	5’-FAM-GCCAGGAA-BHQ-1	315
*rCTSG*	NM_001106041.1	Forward: TGTGGGAAACCCGAGAGAAA Reverse: GGGAGTCAGCAAGTCCCTAATA	5’-FAM-CCAGGGCA-BHQ-1	209
*tRNA* ^*Leu*^	NC_012920.1	Forward: CACCCAAGAACAGGGTTTGT Reverse: TGGCCATGGGTATGTTGTTA	-	107
*β2-microglobulin*	NC_000015.10	Forward: TGCTGTCTCCATGTTTGATGTATCT Reverse: TCTCTGCTCCCCACCTCTAAGT	-	86

### cfDNA isolation

Equal amount of cells were seeded and differentiated on 10 cm Ø cell plates. Conditioned medium was added for 72 h, and then, 6 ml of culture medium was collected and centrifuged to remove cells and large debris. The supernatants were used to extract cfDNA via a cell-free AX DNA kit (054–50; A&A Biotechnology, Poland) according to the manufacturer’s instructions. The amount and purity of the DNA were assessed using spectrophotometry (Nanodrop2000, Thermo Fisher, MA, USA).

### Western blot

Cell lysates were prepared using RIPA lysis buffer (0.5 M Tris-HCl [pH 7.5], 2.5 M NaCl, 2.5% deoxycholic acid, 10% NP-40, and 10 mM ethylenediaminetetraacetic acid [EDTA]) with a protease inhibitor cocktail (Sigma-Aldrich, MO, USA). The lysates were centrifuged for 20 min at 18,000 × *g*. Equal amounts of total protein (30 µg/well) were separated on sodium dodecyl sulfate‒polyacrylamide gels (10‒12%) and electrotransferred to polyvinylidene fluoride membranes. The membranes were blocked for 1.5–24 h with 3.5% nonfat dry milk and incubated with primary antibody overnight at 4 °C. The membranes were then washed and incubated with secondary antibody for 1–2 h. Protein bands were detected using colorimetric reaction for detecting alkaline phosphatase activity (5-bromo-4-chloro-3-indolylphosphate/nitroblue tetrazolium (BCIP/NBT) system) or by chemiluminescence. Band densities were quantified via the Quantity One program (Bio-Rad, CA, USA) and ImageJ. The antibodies used are listed in Table 2.

**Table 2:** Antibodies used for Western blotting and immunofluorescence

Western blot primary antibody	Clonality	Dilution	Source
Cathepsin G	polyclonal	1:2000	ABclonal, A13172
Neutrophil elastase	polyclonal	1:2000	ABclonal, A13015
Proteinase 3	monoclonal	1:2000	ABclonal, A19748
SerpinE1	monoclonal	1:2000	ABclonal, A19096
SerpinB1	monoclonal	1:1000	Santa Cruz Biotechnology, sc-293,462
SerpinA3	polyclonal	1:1000	Invitrogen, PA5-86755
NOX2	monoclonal	1:600	Abcam, ab129068
NOX4	monoclonal	1:600	Santa Cruz Biotechnology, sc-518,092
Actin	monoclonal	1:20000	Sigma‒Aldrich, A5441
Calnexin	monoclonal	1:1000	Santa Cruz Biotechnology, sc-46,669
CD81	monoclonal	1:250	Santa Cruz Biotechnology, sc-166,029
Flotillin-2	monoclonal	1:500	Santa Cruz Biotechnology, sc-28,320
Caspase-3	polyclonal	1:500	Santa Cruz Biotechnology, sc-7148
Western blot secondary antibody	Detection	Dilution	Source
Mouse	alkaline phosphatase	1:2500	Sigma‒Aldrich, A3562
Rabbit	alkaline phosphatase	1:2500	Merck, A3812
Mouse	horseradish peroxidase	1:3000	Sigma‒Aldrich, A3562
Rabbit	horseradish peroxidase	1:3000	Sigma‒Aldrich, A9169
Immunofluorescence primary antibody	Clonality	Dilution	Source
Cathepsin G	polyclonal	1:50	ABclonal, A13172
Neutrophil elastase	polyclonal	1:50	ABclonal, A13015
Proteinase 3	monoclonal	1:50	Santa Cruz Biotechnology, sc-74,534
SerpinE1	monoclonal	1:50	Santa Cruz Biotechnology, sc-5297
SerpinB1	monoclonal	1:30	Santa Cruz Biotechnology, sc-293,462
SerpinA3	polyclonal	1:50	Invitrogen, PA5-86755
Myeloperoxidase	monoclonal	1:50	Santa Cruz, sc-52,707
Immunofluorescence secondary antibody	Fluorophore	Dilution	Source
Mouse	Alexa Fluor 546	1:200	Invitrogen, A11030
Mouse	Alexa Fluor 488	1:200	Invitrogen, A11001
Rabbit	Alexa Fluor 546	1:200	Invitrogen, A11010
Rabbit	Alexa Fluor 488	1:200	Invitrogen, A21441

### Immunofluorescence

The cells were fixed in 4% paraformaldehyde for 20 min, permeabilized in 0.1% Triton-X 100 for 1 min, and blocked in FBSB (2% FBS, 2% bovine serum albumin, and 0.2% fish gelatin) for 1 h. The samples were incubated with primary antibodies diluted in FBSB overnight at 4 °C and then with secondary antibodies (1 h at room temperature). Distribution of F-actin in podocytes was examined by Alexa Fluor™ 594 Phalloidin (Thermo Fisher Scientific, MA, USA). The samples were imaged via a confocal laser scanning microscope (Nikon Ti Eclipse, Tokyo, Japan). The primary and secondary antibodies used for immunofluorescence staining are listed in Table 2.

### Assessment of human NE activity

To measure the active form of human NE, a fluorescent substrate was used [31]. For the activity assay, 20 µl of the substrate ABZ-Met-Pro-Val-Ala-Trp-Glu-Tyr(3-NO_2_)-NH_2_ (final concentration in the test well: 47 µM) was incubated at 37 °C with 10 µl of sample (cell lysate or extracellular medium that was ultrafiltered with an Amicon Ultra Centrifugal Filter, 10 kDa, Merck, catalogue no. UFC5010) that was diluted in 168 µl of assay buffer (100 mM Tris and 500 mM NaCl [pH 7.5]) and 2 µl of dimethylsulfoxide (DMSO) in each measuring well. As a specific activity control, the sample was preincubated for 30 min with 2 µl of Sivelestat, which was dissolved in DMSO, a selective human NE inhibitor [32] (final concentration in the test well: 23 µM), at room temperature in the assay buffer. The change in fluorescence intensity was observed for approximately 3 h (λ_ext_ = 320 nm, λ_em_ = 450 nm). All measurements were recorded on a CLARIOStar instrument (BMG Labtech, Germany) using a Nunc F96 microwell black polystyrene plate (Thermo Fisher, catalogue no. 237107).

### Assessment of human PR3 activity

To measure the biological activity of PR3, a fluorescent substrate with the sequence ABZ-Tyr-Tyr-Abu-Asn-Glu-Pro-Tyr(3-NO_2_)-NH_2_ was used [33]. The activity assay was performed as follows. Assay buffer (155 µl; 100 mM Tris and 500 mM NaCl [pH 7.5]) was added to 10 µl of cell lysate or extracellular medium (ultrafiltrated with an Amicon Ultra Centrifugal Filter, 10 kDa, Merck, catalogue no. UFC5010) in each measuring well. As a specific activity control, the sample was preincubated for 30 min with 15 µl of protinin, which was dissolved in H_2_O, a serine protease inhibitor [34] (final concentration in the test well: 0.29 mM), at room temperature in the assay buffer. Afterward, 20 µl of fluorescent substrate was added (final concentration in the test well: 47 µM) to each well, and fluorescence was measured via a CLARIOStar microplate reader (BMG LABTECH, Ortenberg, Germany) for approximately 3 h (λ_ext_ = 320 nm, λ_em_ = 450 nm) at 37 °C. A Nunc F96 microwell black polystyrene plate (Thermo Fisher, catalogue no. 237107) was used for all measurements.

### Assessment of myeloperoxidase activity

The proteolytic activity of MPO in the biological samples was measured via a myeloperoxidase assay kit (Merck, catalogue no. MAK516) and a Nunc F96 microwell black polystyrene plate (Thermo Fisher, catalogue no. 237107) to measure fluorescence. Initially, 25 µl of cell lysate and 20 µl of the MPO inhibitor (20 × Stock) or 20 µl of H_2_O were added to each measuring well. The samples were incubated for 10 min at room temperature. Afterward, 60 µl of working reagent (60 µl of assay buffer, 1 µl of 0.007% H_2_O_2_, and 1 µl of dye reagent) was added to each well, and fluorescence was measured via a CLARIOStar microplate reader (BMG LABTECH, Ortenberg, Germany; λ_ext_ = 530 nm, λ_em_ = 585 nm) at 37 °C.

### Measurement of oxidative stress and NADPH oxidase activity

Oxidative stress was measured using the 2’,7’-dichlorodihydrofluorescein diacetate (Thermo Fisher, catalogue no. D399) fluoroprobe as described previously with minor modifications [35]. The fluorescence emission of dichlorodihydrofluorescein (DCF) was measured via a spectrofluorometer (Enspire, Perkin Elmer) with excitation/emission wavelengths of 485/525 nm. The results of intracellular oxidant generation are expressed in arbitrary units (AUs). NADPH oxidase activity in podocytes was measured via the lucigenin-enhanced chemiluminescence method [36] with modifications [35]. To measure superoxide anion production, 200 µl of cell homogenate (50 µg protein) was added to 290 µl of PBS buffer containing 1 mM EDTA and 20 µM lucigenin (Sigma-Aldrich, catalogue no. M8010). The assay was initiated by adding 100 µM NADPH (10 µl, Sigma-Aldrich, catalogue no. N5130). Photon emission, in terms of relative light units (RLU), was measured every 30 s for 12 min in a Sirius 2 luminometer (Berthold). No measurable activity was detected in the absence of NADPH. The amounts of superoxide were calculated by integrating the area under the signal curve. These values were compared with a standard curve that was generated using xanthine/xanthine oxidase as described previously [37].

### Extracellular vesicle (EV) isolation

The cells were cultured in RPMI 1640 with 1% P/S and 10% exosome-depleted FBS (Gibco, catalogue no. A2720801) for 72 h. The medium was collected and subjected to subsequent centrifugation steps: 300 × *g* for 10 min, 3000 × *g* for 30 min, 10,000 × *g* for 30 min, and 100,000 × *g* for 2 h. The obtained pellet was resuspended in 0.2 μm-filtered PBS and centrifuged for 2 h at 100,000 × *g* at 4 °C. EV markers were detected via Western blotting. The quantity and size of the EVs were measured via nanoparticle tracking analysis (NTA).

### Nanoparticle tracking analysis (NTA)

The size and concentration of the obtained EVs were determined by NTA using a NanoSight NS300 (Malvern Panalytical, Malvern, UK) that was equipped with a 488 nm laser beam and an sCMOS camera. The data were analysed with NanoSight NTA 3.4 software (Build 3.4.003) with a detection threshold set at 5. The isolated EVs were diluted 250-fold in 0.1 μm-filtered PBS. Videos of the samples were recorded five times for 60 s each at a camera level of 13. Background measurements were conducted three times for 60 s each, with the same settings and PBS used for the samples.

### Transmission electron microscopy (TEM)

Podocytes were fixed in 4% paraformaldehyde and 0.25% glutaraldehyde (Agar) in MTSB buffer for 1 h, postfixed for 15 min in 0.5% osmium tetroxide (Agar), and then gradually dehydrated in ethanol and embedded in EPON resin (Agar). Samples were cut (65 nm) on a Leica UC7 ultramicrotome. For immunolocalization, the samples were blocked with 1% BSA-C (Aurion) in PBS for 30 min, after which the grids were incubated with rabbit anti-NE (1:250), anti-PR3 (1:150), or anti-CTSG (1:150) in PBS overnight at 4 °C. The samples were then rinsed with PBS and incubated with 10 nm gold-conjugated goat anti-rabbit IgG (Sigma-Aldrich, MO, USA; 1:50) for 2 h at room temperature. The samples were then washed and stained with uranyless and Reynold’s lead citrate (Delta Microscopies). Analyses were performed using a Tecnai Spirit BioTWIN transmission electron microscope at 120 kV.

### Activity of caspase 3/7, cell viability and cytotoxicity

Activity of caspase 3/7, cell viability and cytotoxicity was measured using ApoTox-Glo™ Triplex Assay (Promega, WI, USA). Cells was seeded on white Nunc™ 96-Well Optical-Bottom Microplate (Thermo Fisher Scientific, MA, USA) and treated with PMA 100nM for 24 h. Cell viability and cytotoxicity was read using fluorescence at 400Ex/505Em nm and 485Ex/520Em nm and activity of caspase 3/7 was read using luminescence on a multimode plate reader (EnSpire, PerkinElmer, Waltham, MA, USA).

### MTT assay

Cell viability was determined colorimetrically using the MTT assay (catalogue co. M6494, Thermo Fisher Scientific, MA, USA). Podocytes were seeded on 96-well plates, with 7000 cells per well. After treatments with appropriate compounds, the cells were incubated for 4 h with 5 mg/ml MTT solution per well. Formazan crystals that formed in the well were dissolved in DMSO overnight. The optical density was measured at 570 nm, with background at 690 nm, using an EnSpire Multimode Plate Reader (PerkinElmer, MA, USA).

### Albumin permeability of the podocyte monolayer

Transepithelial permeability was assessed by measuring the diffusion of FITC-labeled BSA (Merck, Darmstadt, Germany) across the podocyte monolayer, as previously described by Oshima et al. (2001) with modifications [30, 38]. Podocytes were seeded onto cell culture inserts (BD Biosciences, San Jose, CA, USA) coated with type IV collagen and placed in 24-well plates. After 24 h incubation with PMA, the culture medium was replaced with serum-free medium (SFM) for 2 h, followed by replacement with 0.3 ml of fresh SFM in the upper compartment and 1.5 ml of SFM containing 1 mg/ml FITC-albumin in the lower compartment. After 1 h of incubation, the medium from the upper compartment was transferred to a 96-well plate, and the FITC-albumin concentration was determined by measuring fluorescence at 490 nm using a plate spectrophotometer (ELx808, BioTek, USA).

### Statistical analysis

The statistical analyses were performed using GraphPad Prism 8 software. The Shapiro‒Wilk test was performed to determine whether parametric or nonparametric post hoc tests should be conducted. Statistical analyses were performed via one-way analysis of variance and Student’s *t* test. The data are expressed as the mean ± SEM. Significance was set at *p* < 0.05.

## Results

### Identification of NSPs in human and rat podocytes

**Fig. 1:**
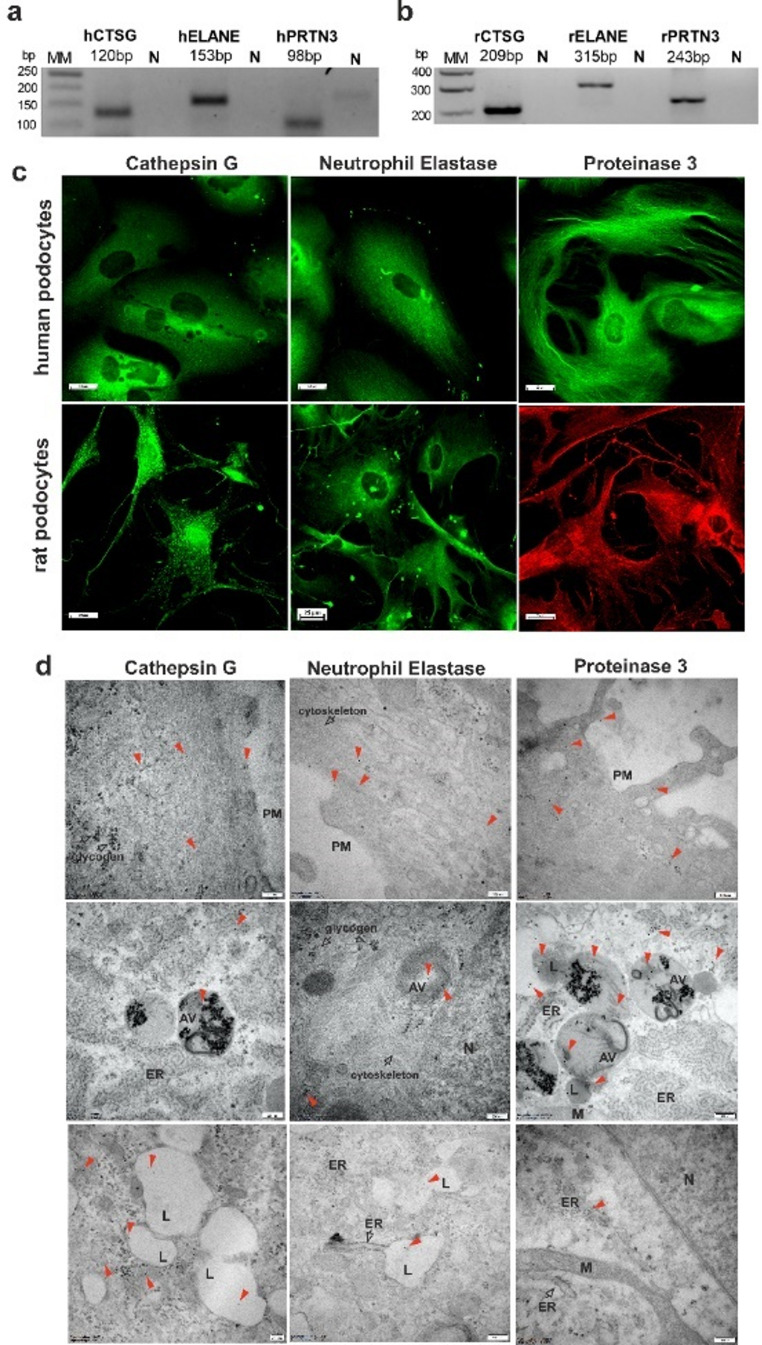
Neutrophil serine proteases expression in immortalized human podocytes and primary rat podocytes. The real-time PCR products, represented in a 2.5% agarose gel, are consistent with the amplified fragments of mRNA that were isolated from human (**a**) and rat (**b**) podocytes. **c** Confocal images of human and rat podocytes that were stained with CG, NE, and PR3. **d** Transmission electron microscopy images of NSPs localization in immortalized human podocytes. PM, plasma membrane; M, mitochondria; ER, endoplasmic reticulum; L, lysosome; N, nucleus; AV, autophagosome vesicle. Red arrows indicate detected proteins

Recently, we demonstrated that podocytes express and secrete CatC [28]. The most well-known function of CatC is the activation of immune cell-associated serine proteinases, such as NSPs. Neutrophil-associated local proteolysis is a common pathogenic phenomenon in inflammatory diseases. Most extracellularly secreted proteinases act as inactive zymogens and require activation. In contrast, NSPs (e.g., elastase, PR3, and CG) and related serine proteinases that are matured by CatC are fully processed and stored as active enzymes in granules of the regulated secretory pathway. In the present study, we identified three NSPs in immortalized human podocytes (h) and primary rat podocytes (r): CTSG, NE, and PR3 (Fig. 1). Using specific primers and hydrolysis probes in real-time polymerase chain reaction (PCR; Table 1), we observed specific bands that corresponded to the calculated product sizes (Fig. 1a, b). For the immunofluorescent detection of NSPs in podocytes, we used the antibodies presented in Table 2 (Fig. 1c). We also identified NSPs in human podocytes via transmission electron microscopy (TEM) with gold-labelled antibodies (Fig. 1d).

### Neutrophil serine protease expression is regulated by inflammatory molecular patterns

**Fig. 2:**
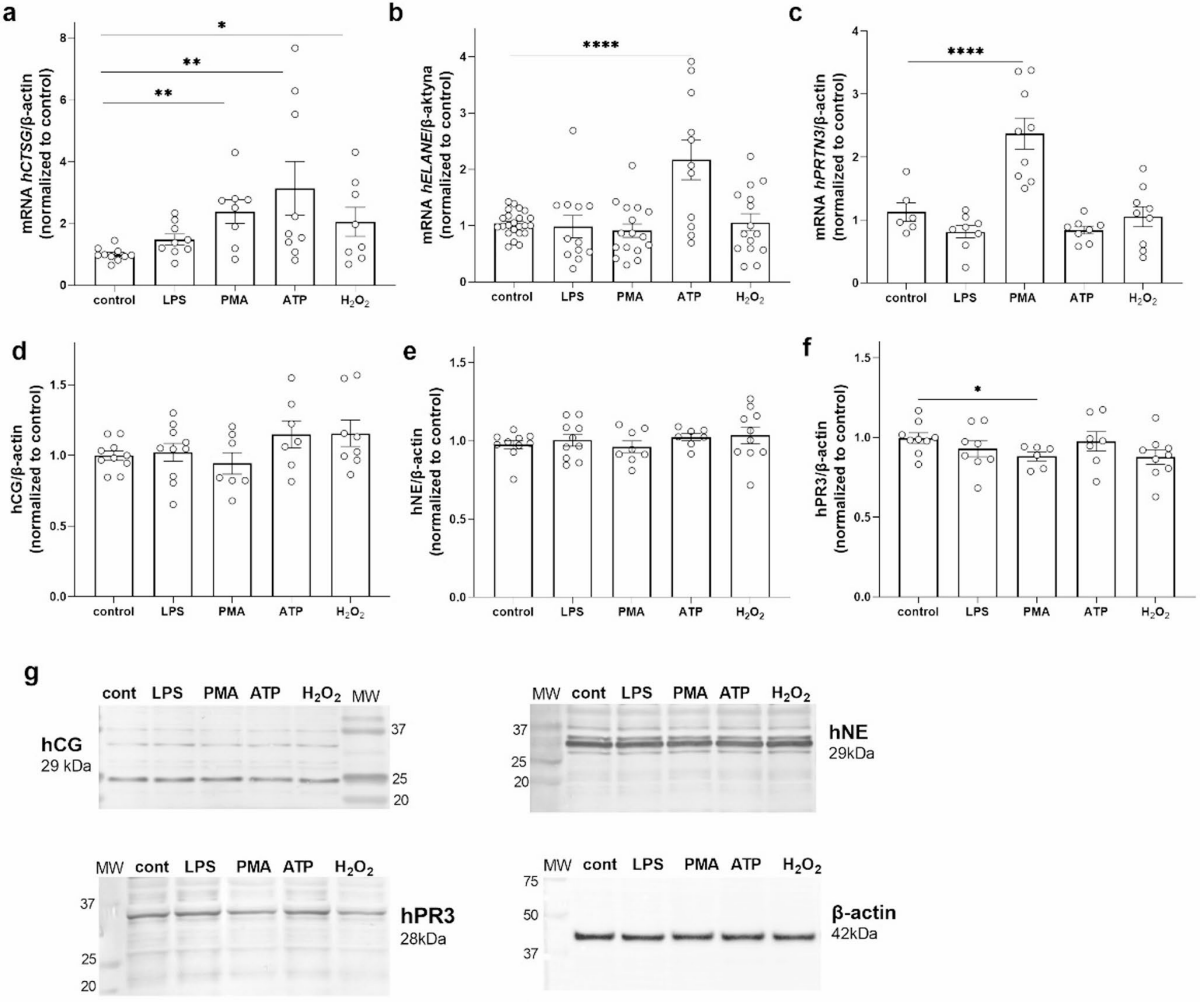
Inflammatory molecular patterns regulate NSP expression. **a** Real-time PCR analysis of hCTSG mRNA expression level. **b** Real-time PCR analysis of hELANE mRNA expression level. **c** Real-time PCR analysis of hPRTN3 mRNA expression level. **d** Western blot analysis of hCG protein expression level. **e** Western blot analysis of hNE protein expression level. **f** Western blot analysis of hPR3 protein expression level. **g** Representative Western blot membranes, with β-actin as a loading control. LPS (1 µg/ml, 24 h), PMA (100 nM, 24 h), ATP (100 µM, 30 min), and H_2_O_2_ (100 µM, 15 min) were used. *p < 0.05, **p < 0.01, ****p < 0.0001

Podocytes are known to exhibit several immune cell-like characteristics that enable a rapid protective response to external threats and metabolic disturbances. Innate immunity is programmed to react immediately to conserved molecules that are released by pathogens (such as PAMPs) and damaged cells of the host (DAMPs). Therefore, we analyzed the effects of selected PAMPs (lipopolysaccharide [LPS], 1 µg/ml, 24 h), compounds inducing DAMP-dependent pathways (adenosine triphosphate [ATP], 100 µM, 30 min; H₂O₂, 100 µM, 15 min), and a potent activator of protein kinase C (PKC)-induced oxidative burst (phorbol 12-myristate 13-acetate [PMA], 100 nM, 24 h) on the mRNA and protein expression of NSPs in human podocytes (Fig. 2). The *hCTSG* mRNA level increased three times after ATP treatment and approximately two times after PMA and H_2_O_2_ treatment (Fig. 2a). We also observed more than a 2-fold increase in *hELANE* mRNA levels under ATP treatment conditions (Fig. 2b) and a two-fold increase in *hPRTN3* mRNA levels after PMA stimulation (Fig. 2c). Interestingly, in cell lysates, we observed only a 12% decrease in hPR3 protein levels after PMA treatment (Fig. 2f), with no significant effects on other NSPs.

### Identification of NSP inhibitors (serpins) and their regulation by inflammatory molecular patterns

**Fig. 3:**
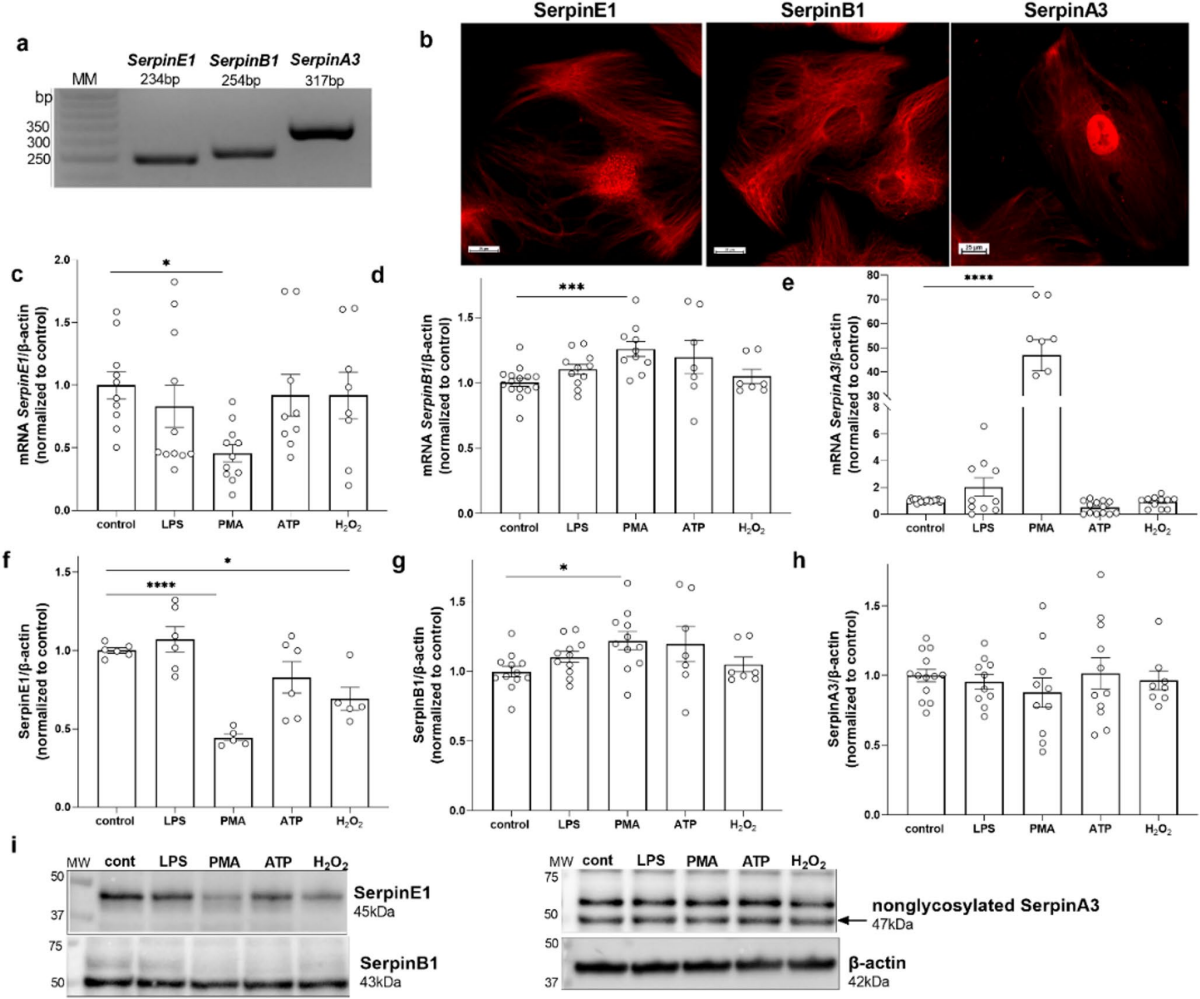
Inflammatory molecular patterns regulate the expression of serpins in podocytes. **a** Identification of the qPCR products via 2.5% agarose gel electrophoresis. **b** Confocal images of SerpinE1, SerpinB1, and SerpinA3. **c** SerpinE1 mRNA expression levels. **d** SerpinB1 mRNA expression levels. **e** SerpinA3 mRNA expression levels. **f** SerpinE1 protein expression levels. **g** SerpinB1 protein expression levels. **h** SerpinA3 protein expression levels. (i) Representative immunoblotting membranes, with β-actin as a loading control. LPS (1 µg/ml, 24 h), PMA (100 nM, 24 h), ATP (100 µM, 30 min), and H_2_O_2_ (100 µM, 15 min) were used. *p < 0.05, ***p < 0.001, ****p < 0.0001

We hypothesized that the physiological balance between proteinases and antiproteinases is required to maintain proper glomerular barrier function. Hence, we next investigated the presence of selected endogenous protease inhibitors in podocytes. We identified three serpins in immortalized human podocytes: SerpinE1, SerpinB1, and SerpinA3. Using specific primers and hydrolysis probes in real-time PCR, we observed specific bands that were consistent with the calculated product size (Fig. 3a). The results of immunofluorescent detection of serpins in podocytes are shown in Fig. 3b. For immunofluorescence, we used the antibodies listed in Table 2. Moreover, we analysed the effects of LPS (1 µg/ml, 24 h), PMA (100 nM, 24 h), ATP (100 µM, 30 min) and H_2_O_2_ (100 µM, 15 min) on serpins by detecting their mRNA (Fig. 3c-e) and protein (Fig. 3f-h) expression. The mRNA levels of *SerpinE1* decreased by 50%, whereas the mRNA levels of *SerpinB1* and *SerpinA3* increased by 25% and by 46-fold, respectively, compared to control, after PMA stimulation. Western blot analyses revealed that PMA and H_2_O_2_ reduced SerpinE1 levels by 50% and 30%, respectively. The protein expression of SerpinB1 increased by 20% after PMA treatment; in contrast, the protein expression of SerpinA3 did not change.

### Effects of LPS, PMA, ATP and H_2_O_2_ on oxidative stress and cell-free DNA release

**Fig. 4:**
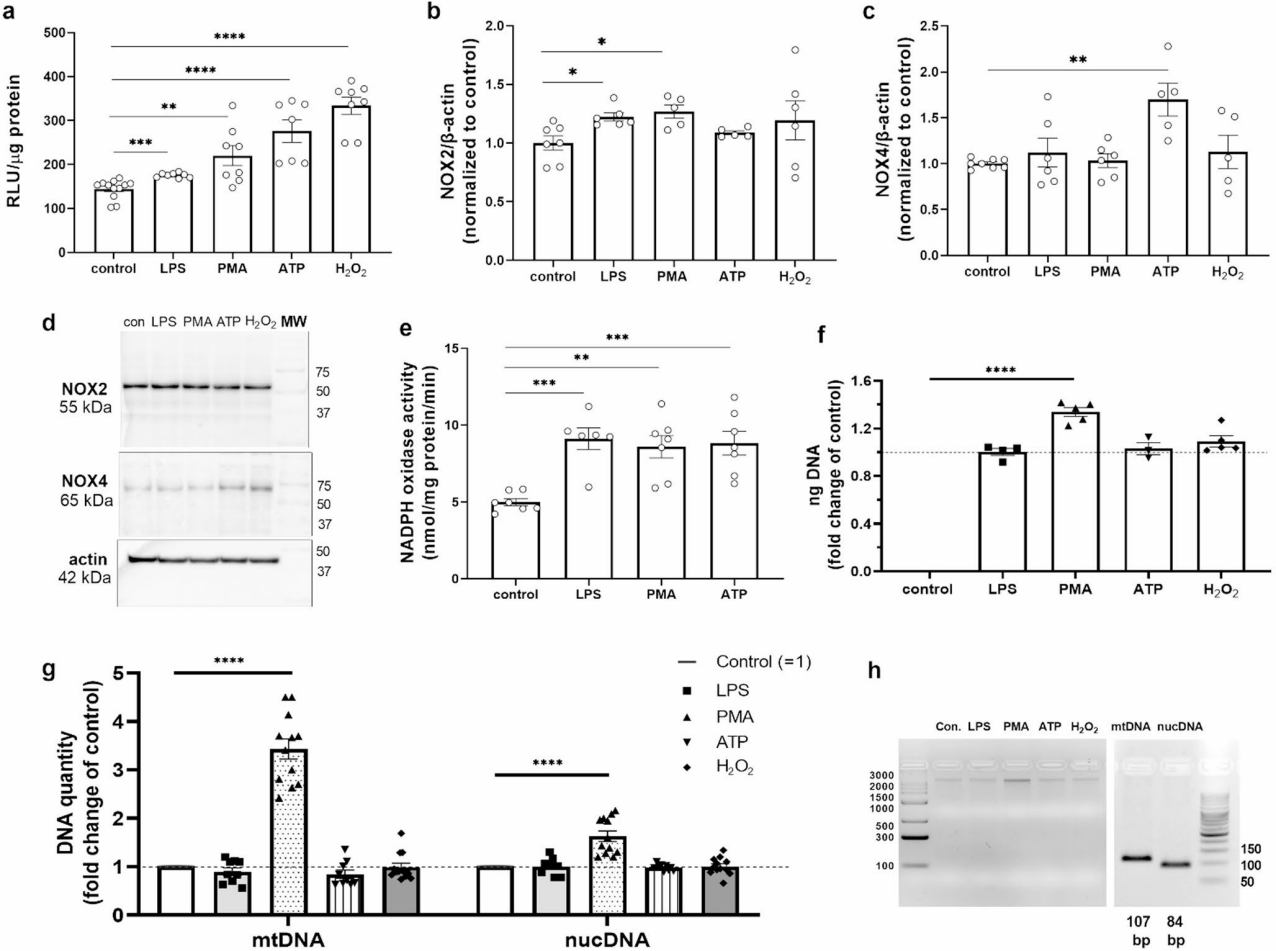
LPS, PMA and ATP induce oxidative stress in podocytes and cfDNA release. **a** Oxidative stress quantity. **b** Western blot analysis of NOX2. **c** Western blot analysis of NOX4. **d** Representative immunoblot membranes. **e** NADPH oxidase activity. **f** Total cfDNA was isolated from 6 ml of podocyte culture medium (72 h; fold change compared with the control). **g** Fold change in cell-free mtDNA and nucDNA secretion after cell treatment with the indicated reagents. **h** Representative agarose gels showing cfDNA quality and size (left) and PCR products (right). LPS (1 µg/ml, 24 h), PMA (100 nM, 24 h), and ATP (100 µM, 30 min) were used. *p < 0.05, **p < 0.01, ***p < 0.001, ****p < 0.0001

To evaluate oxidative stress, we measured ROS production and NADPH oxidase activity in human podocytes after stimulation with LPS (1 µg/ml, 24 h), PMA (100 nM, 24 h), and ATP (100 µM, 30 min). ROS production increased by 20% after LPS treatment, 50% after PMA, and 90% after ATP (Fig. 4a). The cells that were exposed to H_2_O_2_ (100 µM, 30 min) were considered positive controls (Fig. 4a). We also measured the protein expression of two different NADPH oxidase isoforms, NOX2 and NOX4, after PAMP and DAMP stimulation. Western blot analysis of NOX2 and NOX4 expression showed that NOX2 protein expression increased by 20% after PAMP stimulation and that NOX4 protein expression increased by 70% after ATP stimulation (Fig. 4b-d). All the treatments increased overall NADPH activity ~ 2-fold (Fig. 4e). Moreover, PMA significantly increased the amount of cell-free DNA (cfDNA) released from podocytes (by ~ 30% vs. the control) (Fig. 4f). Using real-time PCR, we detected cfDNA of both mitochondrial and nuclear origin (Fig. 4g, h). PMA treatment resulted in an ~ 3.5-fold increase in mitochondria-derived cfDNA and an ~ 50% increase in nucleus-derived cfDNA, indicating that mitochondrial DNA (mtDNA) may play a predominant role in the podocyte response to PMA.

### Podocytes release enzymatically active neutrophil serine proteases, revealing a novel mechanism of podocyte involvement in glomerular function and the immune response

**Fig. 5:**
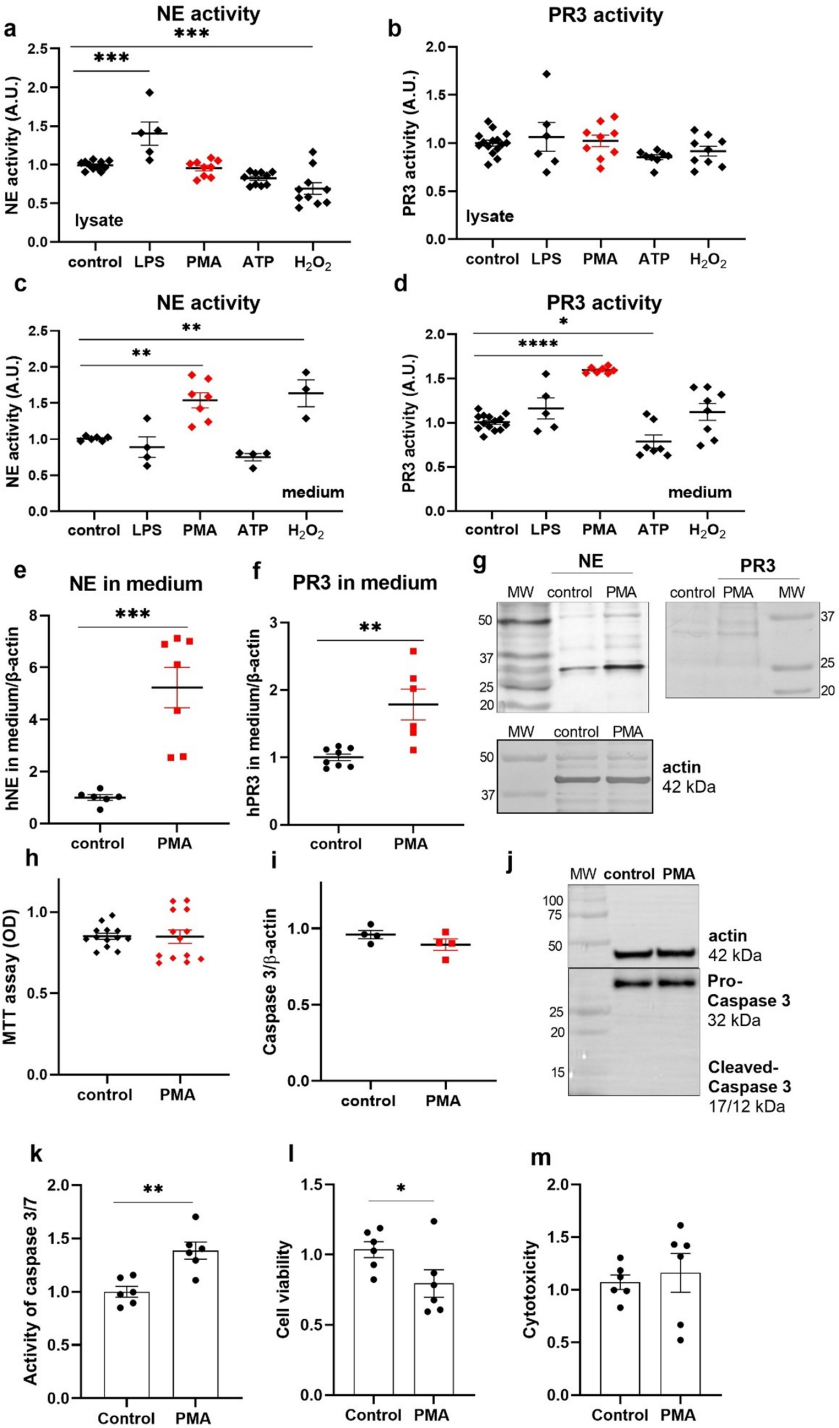
LPS, PMA, ATP and H_2_O_2_ influence intra- and extracellular NSPs activity. **a**, **b** Intracellular activity of NE (**a**) and PR3 (**b**). **c**, **d** Extracellular activity of NE (**c**) and PR3 (**d**). **e**, **f** Influence of PMA on the extracellular levels of NE (**e**) and PR3 (**f**). **g** Representative immunoblot membranes for NE, PR3, and actin. **h** MTT assay. **i** Western blot of caspase 3. **j** Representative immunoblots of procaspase 3 and cleaved caspase 3. **k**-**m** PMA induces apoptotic signalling in podocytes without increasing cytotoxicity. ApoTox-Glo™ Triplex Assay was used to assess apoptosis (k), cell viability (l), and cytotoxicity (m) in podocytes treated with PMA. LPS (1 µg/ml, 24 h), PMA (100 nM, 24 h), ATP (100 µM, 30 min), and H_2_O_2_ (100 µM, 15 min) were used. *p < 0.05, **p < 0.01, ***p < 0.001, ****p < 0.0001

Because neutrophils rapidly release enzymatically active NSPs in response to inflammatory stimuli, we next examined whether podocytes exhibit a similar mechanism of NSP release into the extracellular environment. We examined the effects of LPS, PMA, ATP and H_2_O_2_ on the intra- and extracellular activity of NSPs (Fig. 5). We observed an ~ 40% increase in the intracellular activity of NE after LPS treatment and a 31% decrease after H_2_O_2_ treatment (Fig. 5a); however, we did not observe any changes in the intracellular activity of PR3 (Fig. 5b). In the extracellular medium, we observed a > 2-fold increase in NE activity after PMA treatment and a 60% increase after H_2_O_2_ treatment (Fig. 5c). We also observed an ~ 60% increase in extracellular PR3 activity after PMA treatment and a 19% decrease after ATP treatment (Fig. 5d). In the subsequent steps of the study, we used only PMA, after which we observed the highest effect on the increase in extracellular NSPs activity and release of cfDNA. We showed that PMA increased extracellular NE and PR3 protein levels (Fig. 5e–g). Importantly, we did not observe a decrease in cell viability (MTT assay; Fig. 5h) or induction of apoptosis, measured as the level of cleaved caspase 3 (Fig. 5i, j), in PMA-treated podocytes. To further assess the effect of PMA on podocyte survival pathways, we employed the ApoTox-Glo™ Triplex Assay to simultaneously quantify apoptosis, viability, and cytotoxicity (Fig. 5k-m). PMA stimulation resulted in a significant increase in caspase-3/7 activity compared with control cells (~ 38% increase; *p* < 0.01), indicating activation of apoptotic signaling. Consistent with this observation, a modest but statistically significant reduction in cell viability was observed (~ 20% decrease; *p* < 0.05) following PMA exposure. In contrast, no significant changes in cytotoxicity were detected between PMA-treated and control podocytes, suggesting that PMA induces apoptotic mechanisms without causing overt loss of membrane integrity. Collectively, these results indicate that PMA triggers apoptosis in podocytes while not increasing acute cytotoxic injury.

### Myeloperoxidase (MPO) expression and activity identified in podocytes

**Fig. 6:**
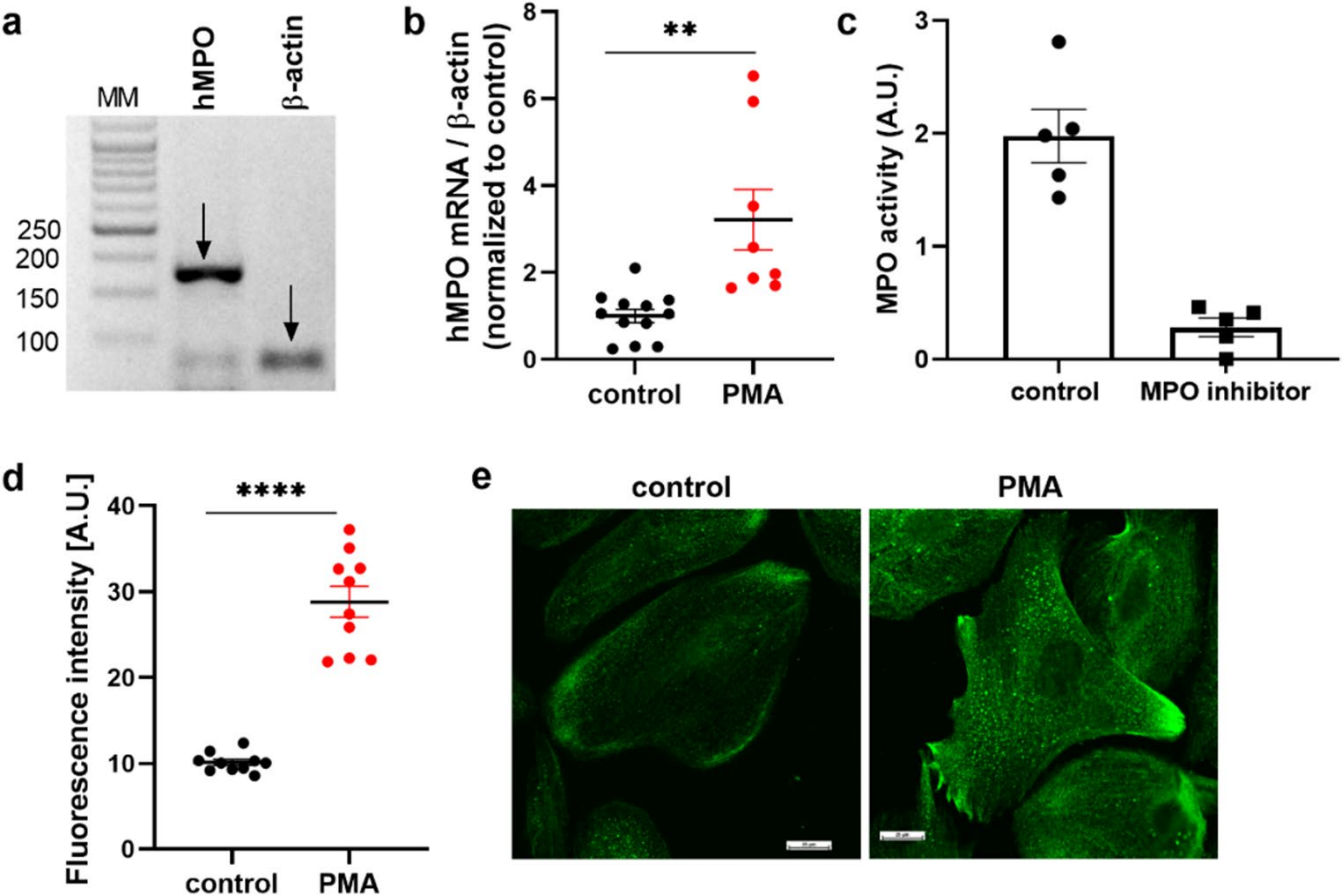
Myeloperoxidase expression, activity, and localization in podocytes. **a** The mRNA expression of MPO was detected in podocytes. **b** Real-time PCR analyses revealed an increase in MPO mRNA expression after PMA treatment. **c** MPO activity was measured via a commercial kit containing an MPO inhibitor. **d** Immunofluorescence labelling of MPO revealed increased expression of MPO in PMA-treated podocytes. **e** Representative images of the immunofluorescent labelling of MPO in podocytes. **p < 0.05, ****p < 0.0001

For the first time, the present study revealed both the presence of mRNA and the enzymatic activity of MPO, an enzyme that is traditionally associated with immune cells, thus shedding new light on its unexpected role (Fig. 6a-c). We found that PMA significantly increased MPO expression (Fig. 6b) and protein levels (Fig. 6d), as indicated by the fluorescence intensity. Moreover, we found that MPO was localized within the cellular granules and that PMA stimulation enhanced granule staining and drove their redistribution toward the perimembranous regions (Fig. 6e). These findings highlight a previously unrecognized regulatory mechanism of MPO in podocytes, suggesting its potential role in glomerular function and immunity.

### PMA increases the abundance of NSPs in EVs

**Fig. 7:**
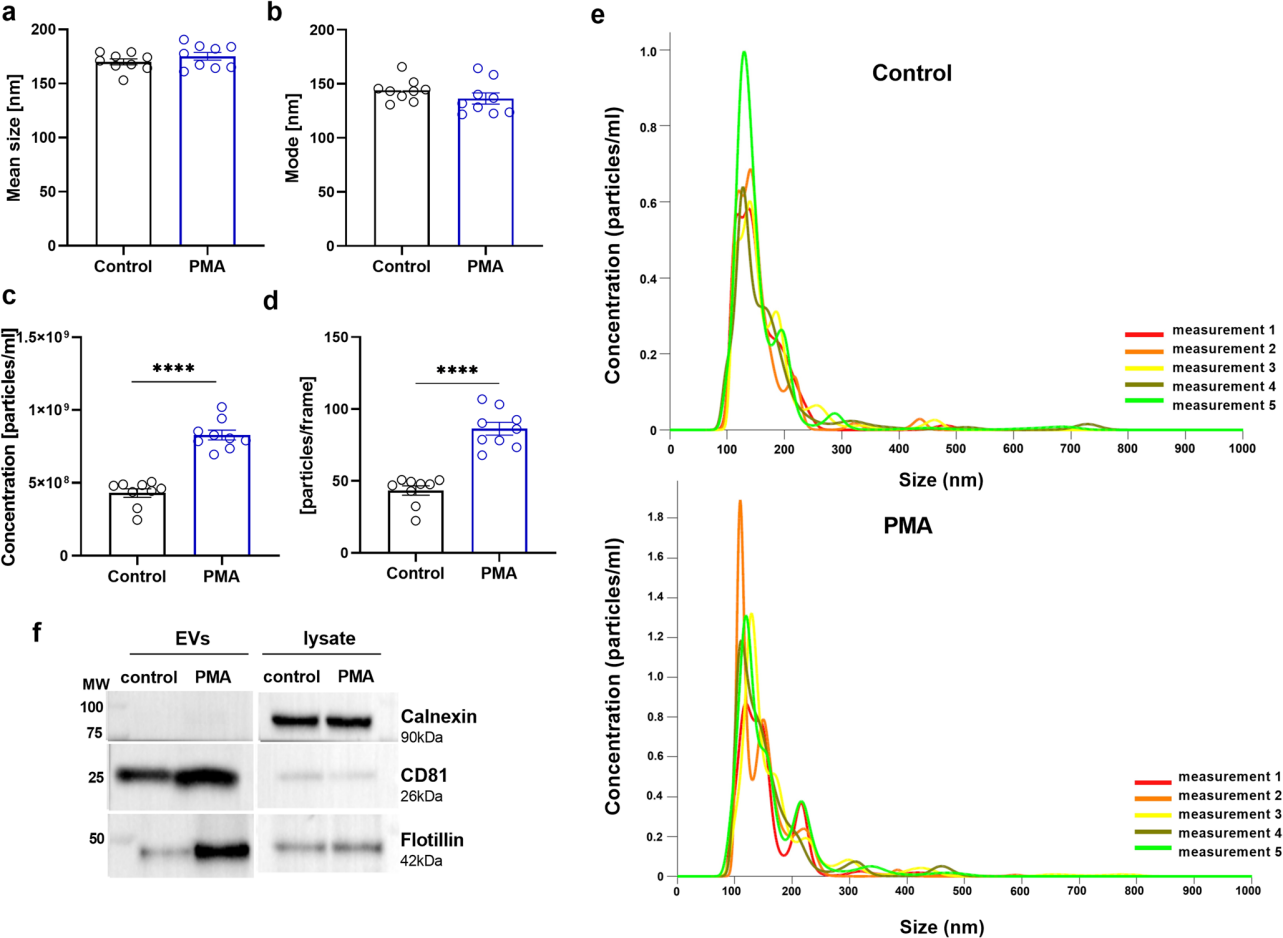
PMA induced EV secretion in human podocytes. **a** Mean sizes of EVs isolated from the culture media of control and PMA-treated cells. **b** Mode values of EVs from control and PMA-stimulated cells. **c** Effect of PMA on EV concentration. **d** Quantity of EVs per frame in NTA analyses. **e** Example of EV size distribution during NTA analysis. **f** Representative immunoblot membranes. PMA (100 nM, 24 h). ****p < 0.0001

**Fig. 8:**
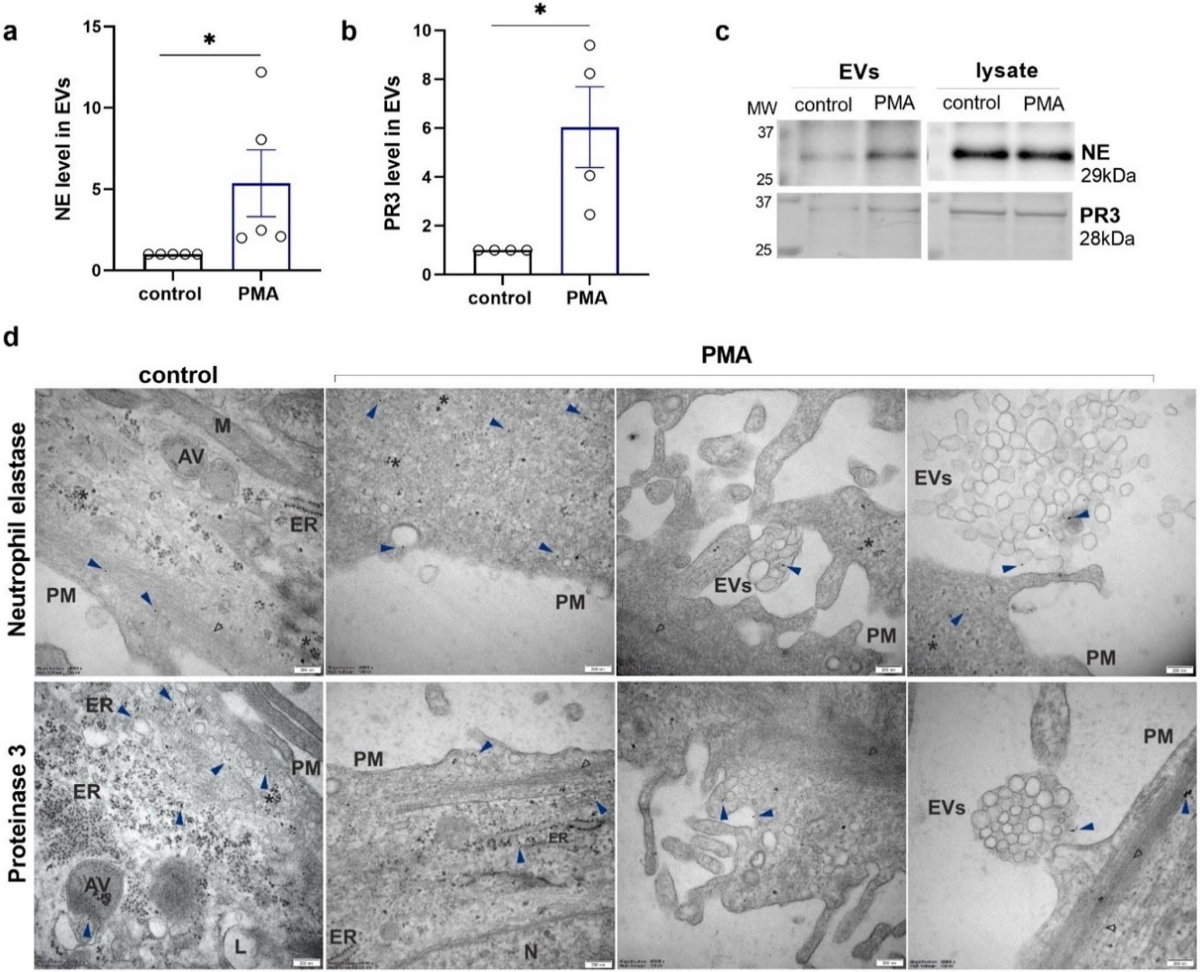
PMA increased NE and PR3 expression in EVs secreted by human podocytes. **a**, **b** Western blot analysis of NE (**a**) and PR3 (**b**) protein expression levels in isolated EVs normalized to the total protein levels detected in the cell lysates. **c** Representative immunoblot membranes. PMA (100 nM, 24 h). *p < 0.05. **d** Transmission electron microscopy images of NE and PR3 in control cells and after PMA stimulation. PM, plasma membrane; ER, endoplasmic reticulum; EVs, extracellular vesicles; M, mitochondrion; L, lysosome; AV, autophagosome vesicle; N, nucleus; *, glycogen. The blue arrows indicate the gold-immunolabelled proteins of interest

On the basis of the above results, especially the extracellular activity of NSPs, we analysed the secretion of EVs by podocytes after PMA stimulation (100 nM, 24 h; Fig. 7). The mean sizes of the isolated EVs were similar for both conditions (170 nm ± 2.6 for the control cells and 175 nm ± 3.06 for the PMA-treated cells; Fig. 7a, b). PMA stimulation significantly increased EV production, the concentration of which was 2-fold greater than that in the control (Fig. 7c, d). In accordance with the MISEV2023 guidelines [39], we characterized the isolated EVs by detecting the expression of marker proteins by immunoblotting (Fig. 7f). We observed an increase in NE and PR3 expression in the EVs secreted by podocytes after PMA treatment (Fig. 8a-c). The presence of NE and PR3 in podocytes and vesicular structures was also confirmed by TEM (Fig. 8d), which highlights their potential role in podocyte function under PMA-induced stress.

### Downregulation of NE expression influences extracellular vesicles release

**Fig. 9:**
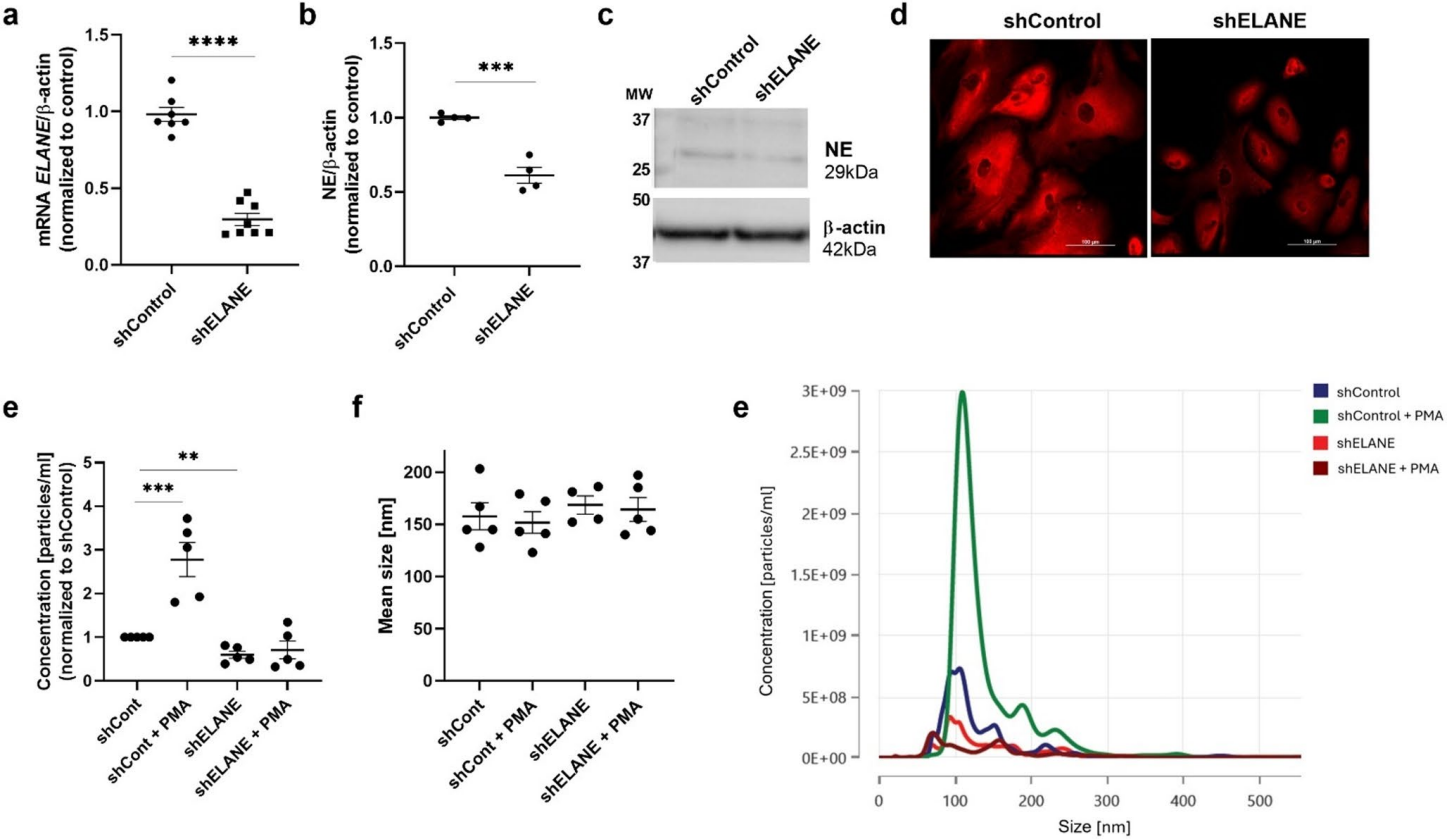
ELANE silencing reduces NE expression and impairs extracellular vesicle release in podocytes. **a** qPCR analysis showing reduction of ELANE mRNA levels in shELANE-transduced podocytes compared to shControl. **b** Quantification of neutrophil elastase (NE) protein by densitometry normalized to β-actin. **c** Representative immunoblot membranes. **d** Immunofluorescence staining for NE illustrating strong cytoplasmic signal in control cells and markedly reduced staining in shELANE podocytes. **e** Nanoparticle tracking analysis (NTA) indicating significantly reduced basal extracellular vesicle (EV) release in ELANE-silenced podocytes. PMA stimulation markedly increased EV secretion in shControl cells but failed to induce a comparable response in shELANE podocytes. **f** Mean EV size distribution showing no major changes across conditions. (g) Example of EV size distribution during NTA analysis. Data represent mean ± SEM; **p < 0.01, ***p < 0.001, ****p < 0.0001 vs. shControl

To further assess the relationship between extracellular vesicle release and NSP mobilization, a podocyte cell line with silenced ELANE expression was generated using lentiviral transduction. Real-time PCR analysis confirmed that ELANE downregulation was successful, achieving a reduction of approximately 70% (Fig. 9a). At the protein level, ELANE silencing resulted in a 39.5% decrease in NE abundance (Fig. 9b, c). Immunofluorescent staining for NE was performed to validate these findings (Fig. 9d).

To further elucidate the functional relevance of NE in vesicular dynamics, we next examined whether decrease of elastase expression affects extracellular vesicle secretion. We observed that podocytes with silenced ELANE expression displayed a markedly reduced release of extracellular vesicles under basal conditions, indicating that NE contributes to the regulation of constitutive vesicle secretion (Fig. 9e-g). Furthermore, ELANE-silenced podocytes did not exhibit an increase in EVs release following PMA stimulation, suggesting that elastase activity is required for the stimulatory effect of PMA on vesicle mobilization.

### ELANE Silencing reduces NSPs activity, oxidative stress and preserves podocyte barrier function and cytoskeleton morphology

**Fig. 10:**
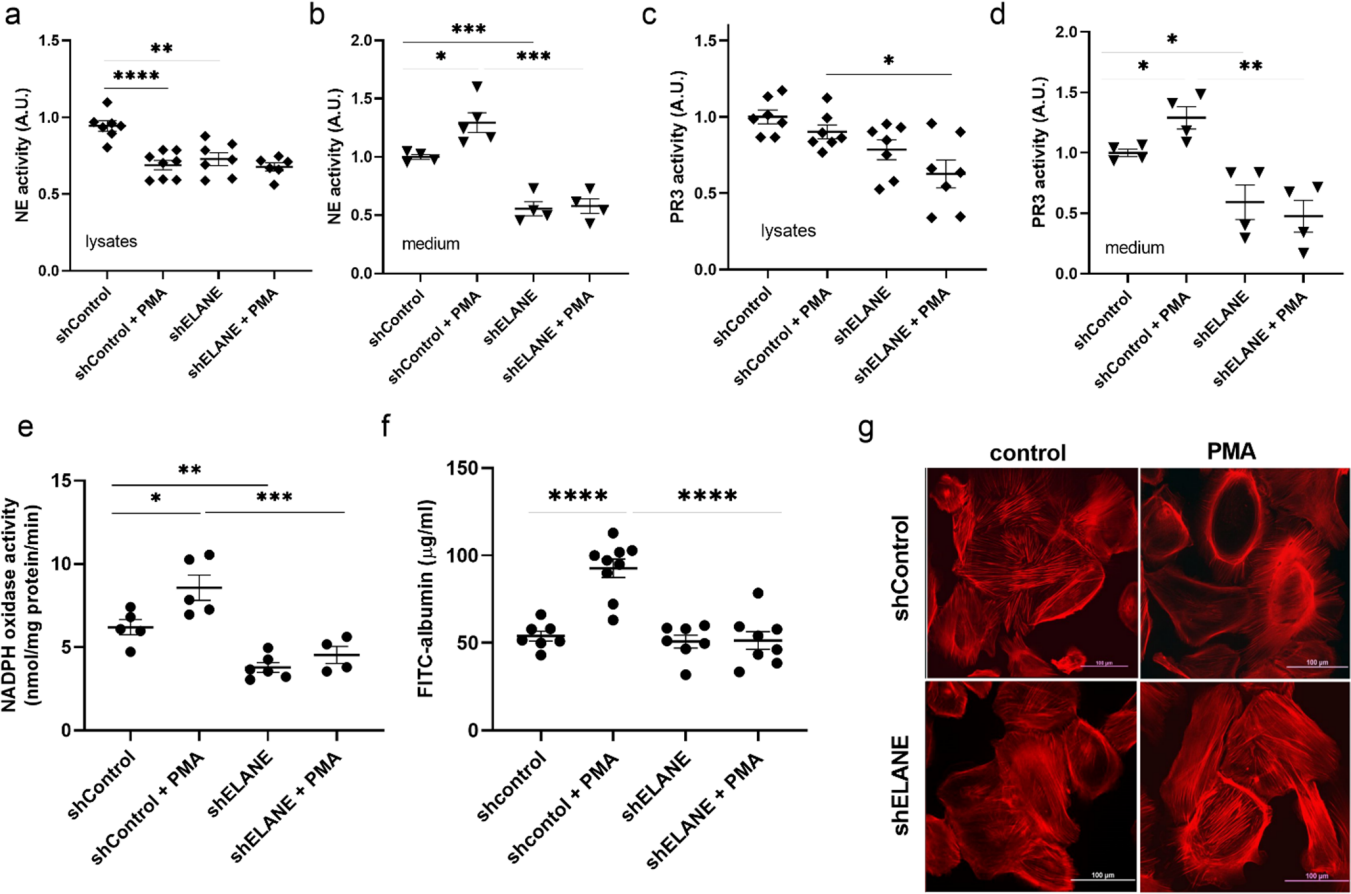
ELANE silencing reduces serine protease activity, oxidative stress, and podocyte injury. NE activity in cell lysates (**a**) and medium (**b**) demonstrating increased activity after PMA stimulation in shControl cells and suppressed activity in shELANE podocytes. PR3 activity in lysates (**c**) and medium (**d**) showing similar trends, with PMA-induced activation in shControl but not shELANE cells. **e** NADPH oxidase activity, elevated in shControl cells after PMA stimulation and significantly lower in shELANE podocytes. **f** FITC-albumin permeability assay showing increased barrier leakage upon PMA stimulation in shControl monolayers, whereas shELANE cells maintain lower permeability. **g** Phalloidin staining demonstrating cytoskeletal disorganization in PMA-treated shControl podocytes, contrasted with preserved actin structure in shELANE cells. Data are mean ± SEM. *p < 0.05, **p < 0.01, ***p < 0.001, ***p < 0.0001

To evaluate the functional consequences of NE depletion, we assessed serine protease activity, oxidative stress, and podocyte barrier integrity. PMA stimulation significantly increased NE activity in shControl podocytes in conditioned media. In contrast, ELANE knockdown markedly reduced NE activity at baseline and abolished PMA-induced enzymatic activation in lysates and extracellular medium (Fig. 10a, b). Proteinase 3 (PR3) activity showed a similar pattern (Fig. 10c, d). NADPH oxidase activity was elevated in shControl cells following PMA exposure but remained low in shELANE podocytes, indicating reduced ROS generation in the absence of NE (Fig. 10e). Functionally, barrier permeability assessed by FITC-albumin flux increased after PMA treatment in shControl cells, whereas shELANE podocytes maintained significantly lower permeability, suggesting preserved barrier integrity (Fig. 10f). Moreover phalloidin staining revealed pronounced cytoskeletal remodeling in shControl podocytes exposed to PMA, while shELANE cells retained organized actin structures, supporting a protective effect of NE silencing (Fig. 10g). Together, these findings identify NE as a mediator of podocyte injury, promoting serine protease activation, oxidative stress, cytoskeletal disruption, and in consequence barrier dysfunction.

## Discussion

The present study provides compelling evidence that podocytes, which are traditionally viewed as terminally differentiated epithelial cells with structural and filtration roles in the glomerulus, exhibit key immune-like properties, including the ability to express and secrete NSPs and their endogenous inhibitors, namely, serpins. This previously unrecognized expression pattern challenges the long-standing notion that NSPs are exclusively derived from hematopoietic lineages and suggests that podocytes may contribute directly to glomerular immune surveillance and inflammatory modulation.

We identified for the first time the presence of PR3, NE, and CG at both the mRNA and protein levels in human and rat podocytes, and we further demonstrated that their expression is responsive to both PAMPs and DAMPs. These findings redefine our understanding of podocyte biology and highlight their potential active role in innate immune responses within the glomerular environment.

Many studies have highlighted the emerging role of podocyte‒neutrophil interactions in the pathophysiology of glomerular inflammation and injury. Kuravi et al.. (2014) reported that podocytes can modulate neutrophil recruitment to inflamed glomerular endothelial cells through the regulation of interleukin-6 (IL-6) signalling [40]. Moreover, neutrophil-derived mediators, such as proteases and ROS, have been shown to induce cytoskeletal rearrangement in podocytes, leading to the disruption of the slit diaphragm and an increase in glomerular permeability [15]. Another study supported the notion that podocytes can function as nonhematopoietic antigen-presenting cells. These cells express MHC class I and II molecules, as well as costimulatory proteins, such as CD80, thereby enabling direct interactions with immune cells and suggesting a potential role in coordinating innate and adaptive immune responses within the glomerulus [41]. Taken together, these studies underscore the dynamic and bidirectional nature of the crosstalk between podocytes and neutrophils.

Clinical studies have shown that peripheral total neutrophils and the neutrophil-to-lymphocyte ratio are also associated with diabetic kidney disease [42, 43]. Additionally, the generation of ROS by NADPH oxidase and the release of granule contents were shown to mediate neutrophil-induced glomerular injury [44, 45]. A high glucose concentration also affects the oxidant‒antioxidant balance in podocytes, resulting in an increase in superoxide anion generation by NADPH oxidase and attenuated metabolism [46]. Activated podocytes release agents that directly stimulate neutrophil chemotaxis and secretory vesicle exocytosis and prime neutrophils for increased generation of ROS [15]. The authors provided evidence of a role for neutrophil granule contents in the disruption of the podocyte cytoskeleton and the loss of podocyte integrity, leading to the development of proteinuria in nephrotoxic nephritis [15]. Our results align with previous observations of PR3 uptake and possible ectopic expression in nonhematopoietic cells [24–26], but we provide new insights into how glomerular epithelial cells might endogenously produce these proteases in response to inflammatory cues.

In addition to gene and protein expression, we present functional evidence that podocytes not only harbor enzymatically active NSPs but also secrete them into the extracellular space in response to inflammatory stimuli, particularly PMA. Our findings indicate that podocytes secrete enzymatically active NSPs, some of which are associated with extracellular vesicles. This observation is consistent with previous studies showing that neutrophils can package NSPs into EVs [47]. However, it should be noted that our current study did not include a direct comparison of protease activity between EV-associated and soluble fractions. Recently, a novel population of exosomes derived from polymorphonuclear neutrophils, which degrade the extracellular matrix via uninhibited elastase activity, was described [48]. Together, these observations indicate that podocytes actively participate in extracellular proteolytic processes through the secretion of NSP-containing EVs, a mechanism reminiscent of neutrophil-derived exosomes. Further investigation is needed to better understand this newly proposed mechanism.

Importantly, we identified three serpins—SerpinE1, SerpinB1, and SerpinA3—as endogenously expressed in podocytes. Their regulation by PAMPs and DAMPs further supports the concept of a tightly regulated protease‒antiprotease balance within the glomerular microenvironment. Notably, SerpinA3 mRNA exhibited a dramatic 46-fold increase following PMA stimulation, although this increase was not accompanied by a corresponding increase in protein levels. These results suggest that complex posttranscriptional mechanisms may modulate serpin bioavailability, potentially to fine-tune podocyte responses during inflammation, and that it may also be secreted into the extracellular environment. This assumption is supported by other studies that identified the abnormal presence of SerpinA3 in the urine of rats with chronic kidney disease [49]. The authors observed the relocalization of SerpinA3 from the cytoplasm to the apical tubular membrane in chronic kidney disease, suggesting its potential secretion into the luminal space under pathological conditions [49]. Another study revealed that in HEK-293 cells that were exposed to H₂O₂ or subjected to starvation, serpinA3 secretion in the culture supernatant increased [50]. These findings suggest that oxidative and metabolic stress are sufficient to promote serpinA3 release, supporting the hypothesis that similar mechanisms may be operative in podocytes under chronic kidney disease conditions.

The present study also highlights a novel role for MPO in podocytes. We demonstrate for the first time that MPO is expressed and enzymatically active in these cells and that its expression and subcellular localization are significantly altered following PMA stimulation. The redistribution of MPO-containing granules towards the plasma membrane suggests a regulated secretory pathway that mirrors classic immune cell behavior. Given the role of MPO in oxidative stress and tissue injury, this observation may have important implications for understanding podocyte dysfunction in glomerular diseases characterized by chronic inflammation.

Podocyte exposure to PAMPs and DAMPs also induced oxidative stress, increased NADPH oxidase activity, and, in the case of PMA, led to the release of mitochondrial cfDNA. The selective release of mtDNA—rather than nucDNA—may serve as a potent DAMP that is able to perpetuate local inflammation. Taken together, these findings suggest that podocytes, through the regulated release of cfDNA and EV-associated proteases, may actively participate in the amplification of glomerular immune responses.

Our findings demonstrate a paradigm shift in podocyte biology, emphasizing the ability of podocytes to act not only as passive targets of injury but also as active participants in the innate immune response. Future studies should investigate the signalling pathways that underlie NSP and serpin regulation in podocytes, the functional consequences of their EV-mediated secretion, and the role of these molecules in vivo. Elucidating how these responses are modulated in disease states may reveal new therapeutic targets to preserve podocyte function and glomerular barrier integrity.

## Data Availability

All datasets generated and/or analysed during the current study are available from the corresponding author upon reasonable request.
